# Added value of computed tomography fractional flow reserve in the diagnosis of coronary artery disease

**DOI:** 10.1038/s41598-021-86245-8

**Published:** 2021-03-24

**Authors:** J. Peper, J. Schaap, J. C. Kelder, B. J. W. M. Rensing, D. E. Grobbee, T. Leiner, M. J. Swaans

**Affiliations:** 1 Department of Cardiology, St. Antonius Hospital, Koekoekslaan 1, 3435 CM Nieuwegein, The Netherlands; 2 Department of Radiology, University Medical Center Utrecht, Heidelberglaan 100, 3584 CX Utrecht, The Netherlands; 3 Department of Cardiology, Amphia Hospital, Molengracht 21, 4818 CK Breda, The Netherlands; 4 Julius Center for Health Sciences and Primary Care, University Medical Center Utrecht, Heidelberglaan 100, 3584 CX Utrecht, The Netherlands

**Keywords:** Cardiology, Cardiovascular diseases, Diagnosis, Medical imaging, Epidemiology

## Abstract

Multiple non-invasive tests are performed to diagnose coronary artery disease (CAD), but all are limited to either anatomical or functional assessments. Computed tomography derived Fractional Flow Reserve (CT-FFR) based on patient-specific lumped parameter models is a new test combining both characteristics simulating invasive FFR. This study aims to evaluate the added value of CT-FFR over other non-invasive tests to diagnose CAD. Patients with clinical suspicion of angina pectoris between 2010 and 2011 were included in this cross-sectional study. All underwent stress electrocardiography (X-ECG), SPECT, CT coronary angiography (CCTA) and CT-FFR. Invasive coronary angiography (ICA) and FFR were used as reference standard. Five models mimicking the clinical workflow were fitted and the area under receiver operating characteristic (AUROC) curve was used for comparison. 44% of the patients included in the analysis had a FFR of ≤ 0.80. The basic model including pre-test-likelihood and X-ECG had an AUROC of 0.79. The SPECT-strategy had an AUROC of 0.90 (p = 0.008), CCTA-strategy of 0.88 (p < 0.001), 0.93 when adding CT-FFR (p = 0.40) compared to 0.94 when combining CCTA and SPECT. This study shows adding on-site CT-FFR based on patient-specific lumped parameter models leads to an increased AUROC compared to the basic model. It improves the diagnostic work-up beyond SPECT or CCTA and is non-inferior to the combined strategy of SPECT and CCTA in the diagnosis of hemodynamically relevant CAD.

## Introduction

The generally accepted reference standard to physiologically assess stenosis-specific ischemia is wire-based fractional flow reserve (FFR)^1–3^. FFR is used for the diagnosis of coronary artery disease (CAD) requiring revascularization. It is defined as the ratio of maximum blood pressure distal to a stenotic lesion relative to normal maximum pressure in the same vessel^4^ It is therefore a useful addition to the anatomical assessment based on invasive coronary angiography (ICA) for the diagnosis of hemodynamically relevant CAD^5^.

As part of a standard protocol to diagnose relevant CAD, multiple non-invasive tests are performed prior to invasive testing in patients with complaints of stable chest pain and a low or intermediate probability of CAD^1,2^. The current non-invasive tests are either limited to anatomical or functional assessments of the degree of coronary artery stenosis and therefore have a low specificity. One of the recommended non-invasive diagnostic tests that uses functional information only is Single Photon Emission Computed Tomography (SPECT). A recent patient-based meta-analysis reported a sensitivity of 0.70 and a specificity 0.78^6^.

Another often-used non-invasive diagnostic test, coronary computed tomography angiography (CCTA) is only based on anatomical information and the ability of assessing the functional severity is lacking. The sensitivity of CCTA is high (87–99%) while the specificity is moderate (61–83%)^7–9^ since CCTA tends to overestimate the lesions severity especially in the presence of calcified plaque. Multiple studies found that visual assessment alone is not sufficient to identify hemodynamically relevant CAD^10,11^.

Recently, a new non-invasive strategy, virtual computed tomography based FFR, that combines both anatomical and functional testing has been developed. The CT-FFR is calculated using parametric lumped models for computational fluid dynamic simulations of the blood flow to estimate invasive FFR^12^. Different algorithms have already been evaluated in multicenter studies demonstrating an improved diagnostic accuracy compared to CCTA alone^13–16^. Diagnostic test results of 0.85 for sensitivity and 0.78 for specificity were reported^8^.

Based on these studies, it is reasonable to assume that CT-FFR might add diagnostic value after a positive or inconclusive CCTA, especially since it does not require additional testing, radiation or contrast medium. Furthermore, it might be an alternative to SPECT and therefore avoid additional use of gamma radiation, additional scan time and no stress testing is needed. To date, the diagnostic performance of CT-FFR has only been described as single test and not in the context of the entire clinical work-up of patients with angina pectoris. Therefore, the aim of this study is to evaluate the added value of CT-FFR beyond the exercise ECG, SPECT or CCTA in patients with intermediate to high pre-test probability of CAD.

## Methods

### Design and study population

This single center study population and the diagnostic work-flow have been previously described by Schaap et al.^17^. Briefly, the study population includes patients having a clinical suspicion of stable angina pectoris and an intermediate to high pre-test likelihood of CAD. Patients were included between 2010 and 2011^17,18^. Exclusion criteria for participating in this study were a history of revascularization for CAD (PCI or CABG) or emergency patients (unstable cardiac condition) since the CT-FFR software is not yet validated in these patients. Patients not in sinus rhythm or with severe heart failure, valvular disease, (possible) pregnancy and contraindications to receive iodinated contrast agent were also excluded for participation in this study. The study conforms to the principles of the Declaration of Helsinki and the regional ethical review board (Medical research Ethics Committees United [MEC-U]) approved the study protocol. Written informed consent was obtained from all patients.

### Diagnostic workflow

All patients underwent an exercise electrocardiogram (X-ECG), stress/rest SPECT, CCS, CCTA and CT-FFR^17^. Imaging acquisition was performed on a hybrid SPECT-CT system, consisting of a gamma camera in combination with a 64-slice CT scanner (CardioMD and Brilliance 64, Philips Medical Systems, Best, the Netherlands). Prior to stress SPECT, X-ECG was performed. Pharmacological stress testing (adenosine at a standard rate of 0.14 mg per kg per minute over 6 min) was used in the cases that patients were unable to perform bicycle stress testing or failed to reach 85% of the predicted maximum heart rate. Rest SPECT was acquired in the case of an abnormal stress SPECT. CCS and CCTA imaging were obtained using a prospectively ECG gated scan acquisition protocol. Patients presenting with a heart rate above 60 bpm were administered intravenous metoprolol until their heart rate was below 60 bmp up to a maximum dose of 20 mg metoprolol. All patients underwent ICA independently of the results of the non-invasive imaging to overcome referral bias. FFR measurements were performed in cases of intermediate stenosis. The interpretation of the diagnostic tests and the reference test was done while blinded for all other tests by a core lab.

### Diagnostic tests

#### Exercise stress ECG

Bicycle stress electrocardiography (X-ECG) was acquired according to the local stepwise protocol^19^. All X-ECG were reviewed in consensus by 2 experienced cardiologist and an abnormal test was defined as a horizontal shift of the ST segment at 80 ms after the J-point of ≥ 0.1 mV in three consecutive beats. The definitions of a non-conclusive test were a decrease of > 30 mm Hg in systolic blood pressure, typical angina pectoris during stress, unable to reach > 85% of the predicted heart rate without (ECG) evidence of ischemia and/or uninterpretable ECG because of an unstable baseline. If none of these criteria were met, the X-ECG was defined as normal.

#### Myocardial perfusion SPECT

Myocardial perfusion SPECT was performed according to the guidelines of the Dutch Society of Nuclear Medicine using a weight-adjusted dose of 400–600 MBq 99mTc-sestamibi. Stress and rest SPECT imaging were interpret using the QGS/QPS software package (Cedars-Sinai Medical Centre, Los Angeles, CA, USA) while blinded for all other tests. Perfusion defects were scored according a five-point system (0 = normal tracer uptake—4 = no tracer uptake). The summed stress score and the summed rest score were calculated and used to define normal, non-conclusive and abnormal perfusion on SPECT as previously described^20^. SPECT classified as non-conclusive was regarded as abnormal in the analysis.

#### CCTA

CCTA was acquired on a Brilliance 64-sliceCTscanner with a prospectively ECG-triggered scan mode according to the society of cardiovascular computed angiography guidelines^21^. A non-enhanced acquisition to calculate the Agatston calcium score (CCS) was performed prior to the CCTA. The CCS was used as continuous variable. The tube voltage ranged between 100 and 120 kVp depending on the BMI of the patients and tube current varied between 600 and 800 mAs. Sublingual nitroglycerine was administered in all patients before image acquisition. All gated images were triggered at 75% of the R-R interval and reconstructed with a slice thickness of 0.8 mm. Obstructive stenosis were scored in a 16-segment model in five categories (0 = 0% area stenosis—5 = 100% stenosis, total occlusion) using the standardized Coronary Artery Disease Reporting and Data System (CAD-RADS)^22^. Segments affected by motion artefacts or blooming due to severe calcification were not assessable and classified as non-conclusive. Subsequently, all segments were summarized to a single per-patient conclusion. An abnormal CCTA scan was defined as at least one segment had a diameter stenosis above 50%. CCTA classified as non-conclusive was regarded as abnormal in the analysis.

#### CT-FFR

3-dimensional (3D) coronary model segmentation and the extraction of the coronary centerlines was performed automatically using a commercially available cardiac application (Comprehensive Cardiac Analysis, IntelliSpace Portal Version 9.0, Philips Medical Systems Nederland B.V., Best, The Netherlands). The coronary lumen segmentation was reviewed in all patients and corrections were made if needed. The effective luminal diameter stenosis (EDS) was measured on the coronary model images by identifying the minimum diameter compared to a reference for all stenosis. The segmented coronary model was used as input for the on-site CT-FFR simulation algorithm prototype (Philips Medical Systems Nederland B.V., Best, The Netherlands)^12^. FFR values were computed by simulating flow in the aorta and in the coronary arteries during simulated hyperemia and pressure ratios were shown as color gradients onto the 3D coronary tree^12,23^. Point estimates of the computed FFR were taken at the lesion of interest, the most severe stenosis at CCTA. A CT-FFR ≤ 0.80 in at least one of the vessels was regarded as an abnormal test.

### Reference test: ICA and FFR

ICA biplane views were acquired from all major coronary arteries using Allura catheterization equipment (Philips Medical Systems, Best, the Netherlands) via femoral or radial artery access. The coronary tree was fully examined for the presence of stenosis according to the 16-segment model as used for the assessment of CCTA. Stenosis above 70% were only assessed visually and considered as functionally significant. Patients suffering from an intermediate stenosis, defined as a diameter reduction between 50 and 70%, or multivessel disease were subsequently assessed by FFR to determine the functional severity of the reduction. The pressure gradient drop across the stenosis was measured during an intracoronary adenosine bolus or continuous intravenous infusion of adenosine at 140 µg/kg/min. The clinical standard of FFR ≤ 0.80 indicating hemodynamically significant stenosis was applied. Vessel-based analysis was performed from which diagnostic accuracy on a per-patient level was determined. Hemodynamically relevant CAD was defined as when at least 1 vessel with an FFR ≤ 0.80 or a diameter stenosis above 70% on ICA.

### Statistical analyses

First, all missing test values, when the percentage of missing values was between 10 and 20%, were imputed (10 times) and combined according Rubin’s rule. Categorical variables are presented as numbers and percentages, while continuous variables are presented as mean and standard deviation in the case of a normal distribution. Skewed continuous variables are presented as median and interquartile range. Diagnostic test statistics as sensitivity, specificity and the predictive values and their 95% confidence intervals were calculated for all non-invasive diagnostic test. The variables were added to the multivariable model in chronological order as in clinical practice, starting with the pre-test likelihood and exercise stress ECG (X-ECG). The tested models include the single and combined results of SPECT, CCS, CCTA and CT-FFR. To assess the ability of the different models to distinguish between patients with and without CAD, the area under the receiver operating characteristic curve (ROC-curve) and the area under the curve (AUC) were calculated (measures of discrimination) and the differences in AUC were compared using the approach of DeLong et al.^24^. The calibration was assessed by means of the Hosmer–Lemeshow goodness of fit statistics and plotting the observed and predicted probabilities of disease for visual assessment. All statistical analyses were performed using R statistical software (www.r-project.org, version 3.4.2).

## Results

**Figure 1:**
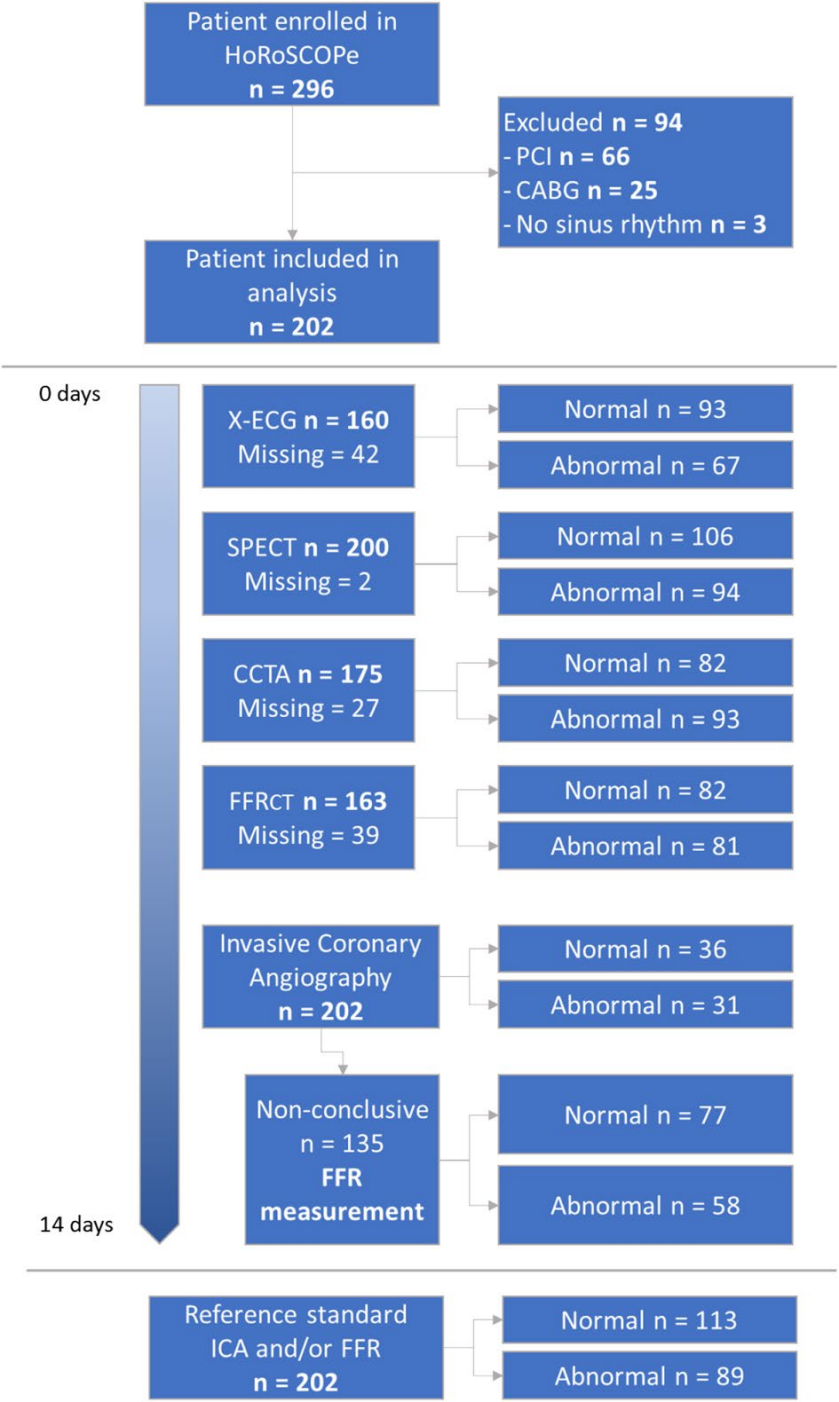
Study enrollment. *CABG* coronary artery bypass graft, *CCTA* coronary computed tomography angiography, *ICA* invasive coronary angiography, *FFR* fractional flow reserve, *CT-FFR* computed tomography fractional flow reserve, *PCI* percutaneous coronary intervention, *SPECT* single photon emission computed tomography, *X-ECG* exercise electrocardiogram.

A total of 296 patients who underwent exercise ECG, SPECT, CCTA, CT-FFR, ICA and FFR were identified to be eligible to be included in the study (Fig. 1). Patients with a prior history of percutaneous coronary intervention (n = 66), Coronary Artery Bypass Grafting (n = 25) and cardiac rhythm other than sinus rhythm (n = 3) were excluded. Therefore, 202 patients were included for statistical analysis. The patient characteristics of the study population are provided in Table 1.

**Table 1:** Baseline and diagnostic test characteristics.

Patient characteristics	n (%)
Age (year) (mean ± sd)	63.1 ± 9.8
Gender (male)	124 (61.4)
BMI (kg/m^2^) (mean ± sd)	27.0 ± 4.2
**Angina pectoris**	
Non-angina	1 (0.5)
A-typical	64 (31.7)
Typical	137 (67.8)
**Risk factors**	
Smoking (ever)	89 (39.1)
Hypertension	131 (64.9)
Dyslipidemia	129 (63.9)
Diabetes mellitus	35 (17.3)
Family history of premature atherosclerosis	121 (59.9)
**Diagnostic tests**	
No missing tests	123 (60.9)
≥ 3 missing test	3 (1.5)
Pre-test likelihood (%) (median (IQR))	87.0 (72.0–94.0)
**Exercise ECG**	
Abnormal	67 (40.9)
Non-conclusive	4 (2.4)
Normal	93 (56.7)
SPECT (abnormal)	94 (47.0)
CCS (median (IQR))	137.0 (0.0–711.5)
CCTA (abnormal)	93 (53.1)
CT-FFR (abnormal)	81 (49.7)
ICA and FFR (abnormal)	89 (44.1)

Continuous variables are reported as means (standard deviation) or medians (interquartile range). Categorical variables are reported as number (%).

*BMI* body mass index, *CCS* coronary calcium score, *CCTA* coronary computed tomography angiography, *ECG* electrocardiograph, *ICA* invasive coronary angiography, *IQR* interquartile range, *FFR* fractional flow reserve, *CT-FFR* computed tomography fractional flow reserve, *SPECT* single photon emission computed tomography.

All non-invasive tests were evaluated individually by means of sensitivity, specificity and accuracy (Table 2). High diagnostic performance test results were found for SPECT and CT-FFR (accuracy: 84.0% and 82.2%), while CCTA and X-ECG yielded lower diagnostic performance (accuracy: 75.4% and 74.4%).

**Table 2:** Diagnostic performance of each diagnostic test compared to the reference standard outcome.

	Hemodynamically relevant CAD						
	Yes (n = 89)			No (n = 113)			Accuracy, % (95% CI)
	N	Sensitivity, % (95%CI)	PPV, % (95% CI)	n	Specificity, % (95% CI)	NPV, % (95% CI)	
X-ECG	66	77.7 (68.2–84.9)	78.5 (69.1–85.6)	94	69.7 (57.8–79.4)	68.7 (56.8–78.5)	74.4 (67.1–80.5)
SPECT	88	83.0 (75.0–88.9)	87.7 (80.1–92.7)	112	85.2 (76.3–91.2)	79.8 (70.6–86.7)	84.0 (78.3–88.4)
CCTA	84	71.4 (61.4–79.7)	79.3 (69.3–86.6)	91	79.8 (70.0–87.0)	72.0 (62.2–80.1)	75.4 (68.5–81.2)
CT-FFR	78	81.2 (71.6–88.1)	84.1 (74.7–92.9)	85	83.3 (73.5–91.9)	80.2 (70.3–86.2)	82.2 (75.6–88.5)

*CAD* coronary artery disease, *CCTA* coronary computed tomography angiography, *CI* confidence interval, *CT-FFR* computed tomography fractional flow reserve, *NPV* negative predictive value, *PPV* positive predictive value, *X-ECG* exercise electrocardiography.

To assess the combined diagnostic value of the non-invasive tests, five multivariable logistic regression models were generated (Table 3). Pre-test likelihood of CAD and the result of the X-ECG were selected for the basic diagnostic model. The basic model significantly improved by individual or combined extensions with SPECT, CCTA and CT-FFR.

**Table 3:** Discrimination and calibration of the models of interest.

		Hosmer–Lemeshow statistic p value	AUC	95% CI	p value DeLong^22^
Model 1	LLH CAD + X-ECG	0.285	0.790	0.726–0.853	< 0.001
Model 2	SPECT	0.247	0.897	0.849–0.944	0.008
Model 3	CCS + CCTA	0.469	0.876	0.829–0.923	< 0.001
Model 4	CCS + CCTA + CT-FFR	0.568	0.929	0.895–0.962	0.398
Model 5	SPECT + CCS + CCTA	0.284	0.944	0.915–0.973	Ref.

*AUC* area under the curve, *CAD* coronary artery disease, *CCS* coronary calcium score, *CCTA* coronary computed tomography angiography, *CI* confidence interval, *CT-FFR* computed tomography fractional flow reserve, *LLH* pretest likelihood, *SPECT* single photon emission computed tomography, *X-ECG* exercise electrocardiography.

**Figure 2:**
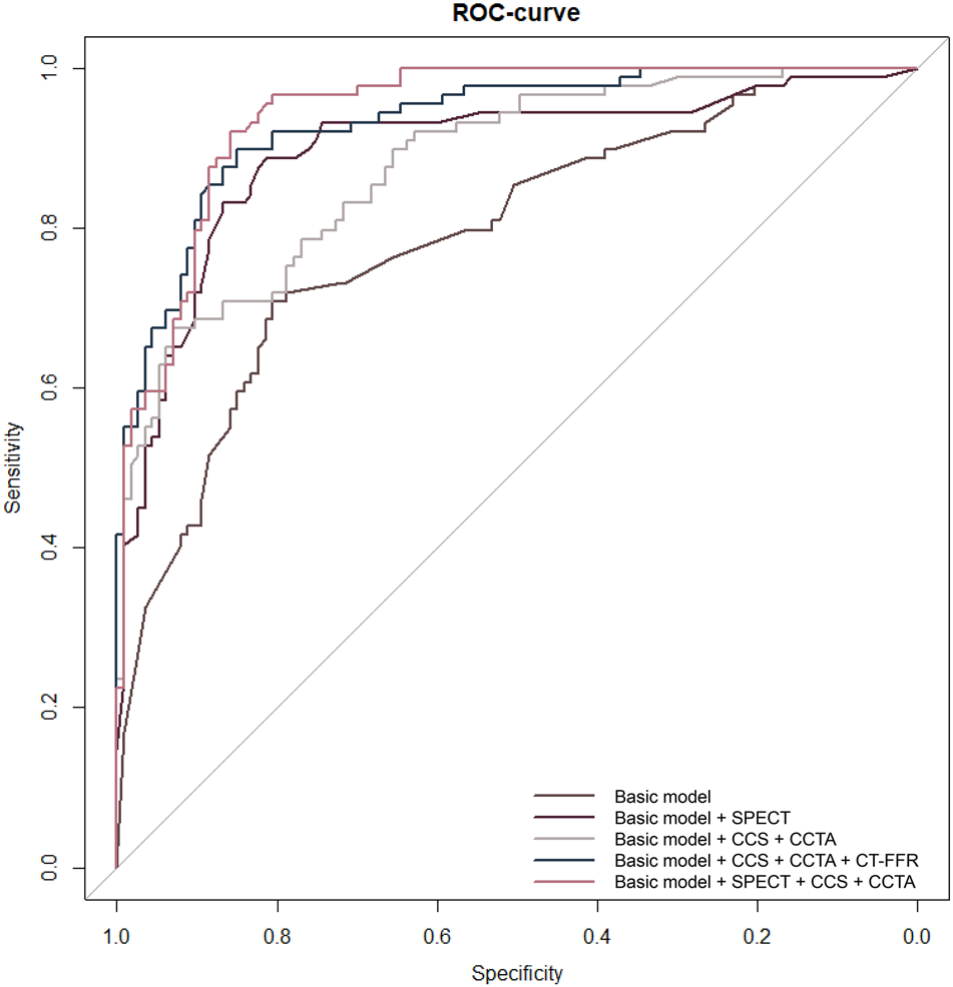
Areas under the Receiver Operator Characteristics curve for all diagnostic model. The basic multivariable model has an AUC of 0.790 and increased to 0.897 upon extension with SPECT. An extension of the basic model with CCTA and CCS increased the AUC to 0.876 and the addition of CT-FFR leads to an AUC of 0.929. The basic model extended with SPECT, CCS and CCTA yielded the highest AUC of 0.94.

**Figure 3:**
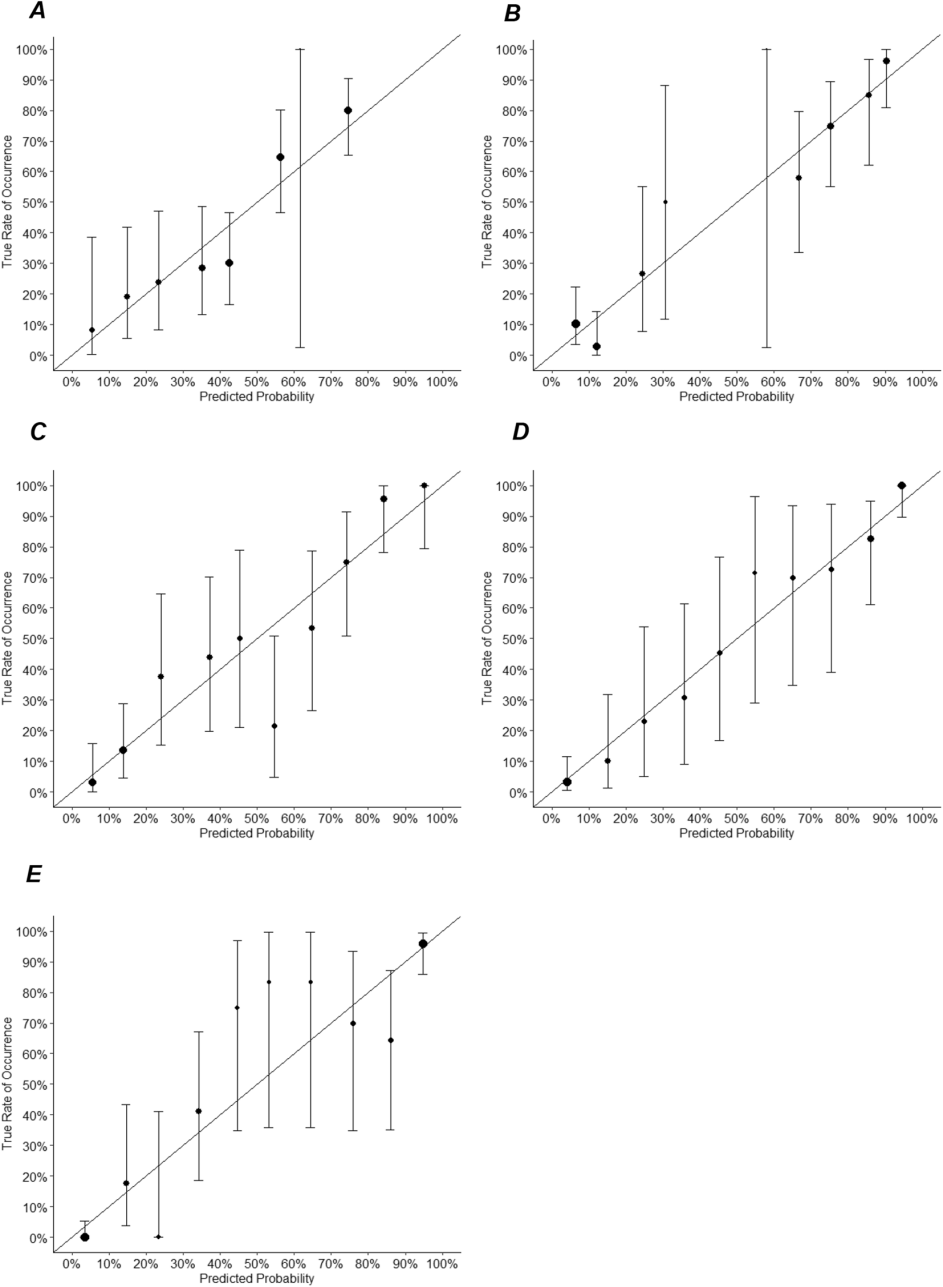
Calibration plots of the diagnostic models. All models show a moderate to good calibration. (**A**) Model 1(basic model) = pretest likelihood of CAD + X-ECG. (**B**) Model 2 = basic model + SPECT. (**C**) Model 3 = basic model + CCS + CCTA. (**D**) Model 4 = basic model + CCS + CCTA + CT-FFR. (**E**) Model 5 = basic model + SPECT + CCS + CCTA.

The basic multivariable model, model 1, had an AUC of 0.790 (95% CI 0.726–0.853, p < 0.001) and increased to 0.897 (95% CI 0.849–0.944; p = 0.008) upon extension with SPECT (model 2). An extension of the basic model with CCTA and CCS (model 3) increased the AUC to 0.876 (95% CI 0.829–0.923, p < 0.001) and the addition of CT-FFR (model 4) led to an AUC of 0.929 (95% CI 0.895–0.962, p = 0.398). The basic model extended with SPECT, CCS and CCTA yielded the highest AUC of 0.94 (95% CI 0.915–0.973) (Fig. 2). All diagnostic models showed a good calibration (Fig. 3), all with p-values above the threshold of 0.05 using the Homer-Lemeshow test of overall goodness of fit (Table 3).

## Discussion

To our knowledge, this is the first study to determine the added value of CT-FFR beyond other commonly performed non-invasive tests such as exercise ECG, SPECT or CCTA in patients with intermediate to high pre-test probability of CAD. We showed that adding CT-FFR improves the AUC compared to the basic model including the pre-test likelihood and X-ECG and it also improves the diagnostic performance beyond SPECT or CCTA. The diagnostic performance of the CCTA and CT-FFR strategy was found to be equivalent in accuracy to the SPECT and CCTA strategy.

The diagnostic performance of CT-FFR as a single test is well described. Celeng et al. analyzed 5330 vessels in their meta-analysis and described a pooled sensitivity of 85% (95% CI 81–90) and a pooled specificity of 73% (95% CI 61–82)^8^. The diagnostic performance of the specific CT-FFR algorithm evaluated in this study has a sensitivity of 91%, specificity of 72% and an accuracy of 78%^23^. Another study performed by Driessen et al. also showed that FFRCT has a higher diagnostic performance than CCTA, SPECT, and PET in a per-vessel analysis. They also showed that PET had a favorable performance in per-patient and intention-to-diagnose analysis^25^.

The diagnostic performance of CT-FFR around the invasively measured FFR cut-off point of 0.80 showed poor agreement with CT-FFR, while slightly broadening the cut-off to CT-FFR values of > 0.82 and ≤ 0.74 provides good agreement^8,26^. However, this is also true for repeated measurements of invasive FFR^8,26^.

### Clinical implications

Widespread implementation of CT-FFR in the current work-up for patients suspected of stable CAD appears feasible. CT-FFR gives additional diagnostic information to the existing pathways, is easy and fast to use, reproducible, and has the potential to be cost-effective^14,27^. CT-FFR adds diagnostic value after a positive or inconclusive CCTA, especially since it does not require additional testing, radiation or contrast medium. The multicenter PLATFORM study demonstrated that up to 61% of the planned ICA can be avoided by using a CT-FFR guided strategy compared to usual care^14^. However, it requires additional operator time of approximately 20 minutes since the segmentation of the coronary centerlines is semi-automatically performed and strongly depends on the scan quality and the amount of calcification present. The CT-FFR guided strategy performs non-significantly different compared to the CCTA-SPECT guided strategy, implicating that SPECT might be replaced by CT-FFR. One of the benefits of not performing an additional SPECT is that additional use of gamma radiation can be avoided. This will save the effective doses of Tc-99m sestamibi for the stress and rest SPECT of 10 mSv^28^. Moreover, no additional scan time is required and there is no stress testing needed. The potential advantages of CT-FFR are twofold: first, it can be used as a gate-keeper to decide whether ICA needs to be performed. Second, increasing evidence shows that the CT-FFR can also be used to guide revascularization^29^. Nevertheless, the revascularization decision does also depend on patient and lesions specific factors as vitality of the patient, characteristics and location of the stenosis.

More research is needed to determine the optimal cut-off value or gray zone for CT-FFR before it can be applied in the clinical work-up. In general, the cut-off value of ≤ 0.80 of invasive FFR is also applied for CT-FFR. It might therefore be preferable to have a gray zone instead of a fixed cut-off value. Cook et al. proposed different scenarios of cut-off values in their meta-analysis to optimize the diagnostic performances of CT-FFR^26^. Besides, Celeng et al. also proposed a gray zone of CT-FFR values between 0.74 and 0.82 ensuring a sensitivity 90% and a specificity of 90%. The accuracy of CT-FFR outside the gray zone was found to be 87%, with a drop to 54% within the gray zone^8^. These diagnostic performance measurement are estimated mainly based on HeartFlow FFR-CT software analyses, which is not used in this study. Nevertheless, the accuracy of Philips CT-FFR software as used in this study seems to be comparable to the performance of HeartFlow FFR-CT^8^.

### Limitations

There are several limitations to our study, including the retrospective character of the data analyses. Although the data collection was performed as part of prospective Horoscope study^17^, we had to deal with missing test results mainly for X-ECG and CT-FFR. To limit the bias caused by the missing data, multiple imputations were performed, but we are aware that missing data hardly occur at random.

Another limitation of this study is that all CCTA examination were performed on older 64-slice scanners. Newer generation CT scanners are likely to yield better results. Technical characteristics that could impact the diagnostic performance of both CCTA and CT-FFR negatively are image artefacts as cardiac and respiratory movement, low contrast, tachycardia or arrhythmia leading to a stair-step artefact, phase misregistration and blooming caused by severe calcification^30^. Since diagnostic accuracy depends on imaging quality, we expect that using current state of the art CT systems will increase the diagnostic accuracy. A third limitation can be found in the selective application of the reference standard. FFR is used as reference standard for vessels with an intermediate stenosis on ICA and have been measured in 67% of the patients. For the remainder of the patients, a threshold of > 50% stenosis was applied as reference standard for hemodynamically relevant CAD. Diagnosing hemodynamically relevant CAD based on ICA is known to be less accurate and might therefore lead to partial verification bias^6^. Last of all, we did not study did not study the performance of stress echo, PET, or stress CMR for the diagnosis of CAD and therefore we are unable to compare these non-invasive tests with CT-FFR^25^.

## Conclusion

On-site CT-FFR based on patient-specific lumped parameter models improves the AUC compared to a work-up strategy of X-ECG alone and it also improves the diagnostic performance beyond SPECT or CCTA. Moreover, a CT-FFR guided strategy is non-inferior to a SPECT and CCTA strategy in terms of discrimination for the diagnosis of hemodynamically relevant CAD. These results imply that a SPECT guided strategy could be replaced by a CT-FFR guided strategy.
